# Genome Sequence of the Pathogenic Bacterium *Vibrio vulnificus* Biotype 3

**DOI:** 10.1128/genomeA.00136-13

**Published:** 2013-04-18

**Authors:** Yael Danin-Poleg, Sharona Elgavish, Nili Raz, Vera Efimov, Yechezkel Kashi

**Affiliations:** Faculty of Biotechnology and Food Engineering, Technion-Israel Institute of Technology, Haifa, Israela;; Bioinformatics Knowledge Unit, Lorry I. Lokey Interdisciplinary Center for Life Sciences and Engineering, Technion-Israel Institute of Technology, Haifa, Israelb

## Abstract

We report the first genome sequence of the pathogenic *Vibrio vulnificus* biotype 3. This draft genome sequence of the environmental strain VVyb1(BT3), isolated in Israel, provides a representation of this newly emerged clonal group, which reveals higher similarity to the clinical strains of biotype 1 than to the environmental ones.

## GENOME ANNOUNCEMENT

*Vibrio vulnificus* is an aquatic bacterium and an important human pathogen (1–4). Strains of *V. vulnificus* are biochemically divided into three biotypes. The highly virulent biotype 3, initially isolated in 1996 (5), appears to be geographically restricted to Israel and found to be rather clonal (6–8), although discrimination was achieved among strains, enabling epidemiology studies (9).

Here we describe the draft genome sequence of the environmental *V. vulnificus* biotype 3 strain VVyb1(BT3), isolated from *Tilapia* fish in 2004 (7, 9) and subjected to whole-genome shotgun sequencing.

The genome complexity of VVyb1(BT3), derived from the high rates of horizontal gene transfer in the *Vibrio* species (10–12) and the presence of multiple repetitive regions, required implementation of a multistep strategy in sequencing and in the bioinformatics analysis. Three different libraries, with average insert sizes of 200 bp, 4,500 bp, and 455 bp, were prepared. The first two libraries were sequenced using Illumina Genome Analyzer IIx, and the third was sequenced using Illumina HiSeq 2000, generating 37,426,728 33-bp paired-end reads, 18,820,750 36-bp mate-pair reads, and 164,399,024 100-bp paired-end reads, with coverages of 215×, 118×, and 2,864×, respectively. All reads of the two first libraries were *de novo* assembled simultaneously with different insert lengths, providing 306 contigs of >500 bp using Velvet 0.7.54 (13). The third library was *de novo* assembled with CLCbio Genomics workbench 5.0 (CLC Bio, Denmark) to generate 470 contigs of >500 bp, using reads with a minimum quality of 30 for each base. The contigs of the third library’s assembly were contiguated (aligned, ordered, and oriented) using ABACAS (14), with *V. vulnificus* CMCP6 (10) serving as a reference genome for chromosomes 1 and 2 and YJ016 for the plasmid (15), to produce 3 scaffolds that split into 95 contigs. This set of contigs was then merged with the assembly of the first two libraries using Minimus2 (AMOS project), providing 69 contigs and numerous singletons that have undergone ABACAS analysis, generating 3 scaffolds plus 93 contigs. Cleaning out sequence redundancy left 84 contigs and 3 scaffolds, resulting in 140 contigs after splitting, with an *N*_50_ of 230,903 bp and the longest contig of 644,564 bp.

The genome of VVyb1(BT3) consists of 2 chromosomes and a plasmid (5.74 Mbp; 46.7% G+C content). A total of 5,361 coding sequences (CDS), 6 rRNAs, and 76 tRNAs were predicted and annotated by the RAST annotation server (16). Of the 5,361 CDS, 217 are unique to VVyb1(BT3) and have low/no similarity (BLASTn, filtering below 85% identity and 80% query coverage) to sequences in the known *V. vulnificus* genomes, and the majority (65%) were annotated as encoding hypothetical proteins. These genes were probably acquired horizontally from other bacterial species sharing the same ecological niche, such as other *Vibrio* species and *Pseudomonas* species. Of the unique genes, 116 show no sequence similarity to any of the known sequences in the NCBI nr database (BLASTn: query coverage, <30%, and E value, >10^–10^). Since biotype 3 is highly clonal, the VVyb1(BT3) genome provides representation of this group, contributing to the understanding of the evolution of this human pathogen.

### Nucleotide sequence accession numbers.

The draft genome sequence for this project has been deposited at DDBJ/EMBL/GenBank under the accession number AOCM00000000. The version described here is the first version, AOCM01000000.
